# Understanding facilitators and barriers to adherence in home-based pulmonary rehabilitation for chronic obstructive pulmonary disease patients using the Health Belief Model: a qualitative study

**DOI:** 10.3389/fpubh.2025.1553744

**Published:** 2025-05-20

**Authors:** Qiuxuan Zeng, Wenli Chen, Siyi Xu, Xu Yang, Dandan Xue, Yuhang Peng, Xiaohong Lin, Yingwen Yu, Huixin Huang, Ping Huang, Min Dong, Jiaying Li

**Affiliations:** ^1^The First Affiliated Hospital of Guangzhou Medical University, Guangzhou Institute of Respiratory Health, Guangzhou, China; ^2^School of Nursing, Guangzhou University of Chinese Medicine, Guangzhou, Guangdong, China; ^3^Li Ka Shing Faculty of Medicine, School of Nursing, The University of Hong Kong, Hong Kong SAR, China; ^4^School of Nursing, Johns Hopkins University, Baltimore, MD, United States

**Keywords:** chronic obstructive pulmonary disease, home-based pulmonary rehabilitation, adherence, Health Belief Model, facilitators, barriers, qualitative research

## Abstract

**Background:**

Home-based pulmonary rehabilitation (PR) is a key non-pharmacological intervention for chronic obstructive pulmonary disease (COPD), but poor adherence limits its effectiveness. The factors influencing adherence, particularly from the patients’ perspective, are not well understood. This study aims to identify the factors that promote or hinder adherence to home-based rehabilitation in COPD patients, to inform more effective and personalized strategies.

**Methods:**

This study guided by Health Belief Model (HBM), and data were collected through semi-structured in-depth interviews with eligible COPD patients during May and July 2024. Deductive thematic analysis approach was used to analyze the data.

**Results:**

Eighteen patients were recruited through purposive sampling. Two main themes emerged-facilitating health beliefs and challenges- along with five subthemes under the HBM framework: perceived threats, perceived benefits, cues to action, self-efficacy, and perceived barriers. In total, 28 categories were identified (21 facilitators, 7 barriers), including 11 newly discovered facilitators and one new barrier. Newly identified facilitators included physical limitations and fatigue, emotional distress, perceived ineffectiveness, time constraints, caregiving responsibilities, lack of support, cost, poor exercise conditions, reduced medical dependence, self-awareness of functional decline, attention-seeking behaviors, environmental cues, mental resilience, and religious beliefs. The new barrier related to environmental limitations and poor exercise conditions.

**Conclusion:**

This study underscores facilitators and barriers influencing home-based PR adherence within the HBM framework. Facilitators include broad factors related to perceived benefits, cues to action, self-efficacy, and perceived threats, while barriers encompass physical limitations, time constraints, and insufficient support. Flexible, personalized programs and greater family involvement can enhance adherence, improve long-term outcomes, and mitigate the rising burden of chronic diseases in aging societies.

## 1 Introduction

Chronic obstructive pulmonary disease (COPD) affects 212.3 million people globally and causes 3.3 million deaths annually (1). In China, it ranks among the top five causes of death (2). COPD is prone to acute exacerbations (3), severely affecting patients’ health and quality of life (4, 5). Rehabilitation is the most effective strategy for symptom improvement (6), but its management is challenging due to an aging population. The prevalence exceeds 21.4% in individuals over 60 and increases with age (7). Older adults patients often have low compliance with medication and rehabilitation, resulting in worse outcomes and more readmissions (8, 9). Up to 80% of COPD patients have at least one comorbidity, with nearly half having three or more, complicating rehabilitation (10, 11). Multimorbidity also increases the financial burden, with annual costs reaching $11,164 per person (12). In China, despite efforts to strengthen primary healthcare, 26 provinces lack sufficient rehabilitation staff, limiting availability (13, 14). In response, home-based pulmonary rehabilitation (PR), requiring minimal resources (15), has shown comparable outcomes to center-based PR (16) and is gaining increasing attention.

Home-based PR has been shown to provide significant benefits for COPD patients, including reduced fatigue, improved exercise capacity and enhanced quality of life (17–19). It is also low-cost, easy to access, and effective in reducing healthcare resource consumption (20). However, adherence to home-based PR remains low, at only 51% (20). The factors influencing adherence are complex, including positive aspects like increased flexibility, personalized care, and reduced travel burdens (21), as well as negative factors such as poor cognition, low motivation (22), insufficient social support (23), and inadequate rehabilitation planning (24). In China, adherence is particularly challenging. 47.5% of older adults are empty-nesters, limiting supervision (25). Limited medical resources in underdeveloped areas and high out-pocket costs in remote western regions hinder continuous rehabilitation guidance (26, 27). Therefore, understanding the factors influencing adherence to home-based PR in China is crucial for developing targeted interventions to improve patient outcomes, enhance quality of life, and reduce healthcare costs.

The Health Belief Model (HBM) is widely used in public health, especially in chronic disease management (28, 29). It explains how individuals’ perceptions of disease severity, susceptibility, benefits, barriers, and self-efficacy influence health behavior (30). These factors are interconnected: individuals who perceive greater severity and susceptibility may be more motivated to act if they believe they can succeed (self-efficacy) and see the benefits outweighing barriers (31, 32). Cues to action, such as healthcare reminders, can further trigger behavior change (33). HBM is particularly valuable for this study, as it helps explain why patients may or may not adhere to PR based on their beliefs, such as whether they think home-based PR will improve their condition and whether they feel confident in their ability to follow through. In China’s complex healthcare context, this research is essential for identifying unique barriers and facilitators, ultimately improving engagement in home-based PR and providing more effectively strategies to enhance patients’ rehabilitation outcomes and quality of life.

This study aims to identify the facilitators and barriers to adherence in home-based PR among COPD patients through a qualitative approach grounded in the HBM. By identifying positive factors that motivate adherence and the challenges that hinder participation, we expect to provide empirical and theoretical support to help policymakers and COPD service providers develop effective rehabilitation programs and strategies that reduce healthcare burdens and improve quality of life for COPD patients.

## 2 Methods

### 2.1 Design

This study employed a qualitative approach, guided by HBM, to explore the facilitators and barriers to adherence in home-based PR among COPD patients.

### 2.2 Setting and participants

Participants were recruited through purposive sampling to ensure diversity in gender, age, and adherence levels. Eligible participants met the following inclusion criteria: (1) COPD patients who already participated in home-based PR for at least three months; (2) capable in understanding and communication; and (3) willingness to engage in interviews. Exclusion criteria included: (1) serious mental illness impacting data reliability; (2) inability to speak or understand Chinese; and (3) language or hearing impairments that would hinder interview participation. The absence of new themes and data saturation guided the determination of the sample size.

### 2.3 Research approach and paradigm

This study adopted a postpositivist paradigm, emphasizing systematic, rigorous methods to approximate objective reality while acknowledging researcher subjectivity and theoretical limitations (34). This approach was well-suited to examining facilitators and barriers to home-based PR adherence among COPD patients, allowing a structured yet flexible exploration of participants’ perceptions, experiences, and behaviors within the HBM framework (35).

### 2.4 Researcher characteristics and reflexivity

The interviewer team comprised two interviewers: S. Xu, an experienced registered nurse specializing in COPD care and PR, with training in qualitative research, and J. Li, Ph.D. in nursing, with advanced expertise in qualitative research methodologies. Their clinical expertise and research skills enabled a deep understanding of the patients’ needs, enhancing the quality and depth of the interviews. Specifically, before the interviews, the primary interviewer (S. Xu) reflected on her role to remain neutral and avoid imposing her views. During the interview, she clarified patients’ understanding and feelings to ensure accurate interpretation. In data analysis, she reviewed and compared text segments to ensure consistency and coherence in her interpretations. J. Li provided guidance on maintaining research rigor throughout the interviews and analysis, ensuring that the process remained grounded in the researchers’ experiences.

### 2.5 Data collection

Data were collected through in-depth semi-structured interviews conducted between May and July 2024. The interview guide included questions focused on perceived susceptibility and severity of COPD, perceived benefits of PR, perceived barriers to adherence, and factors motivating continued participation in PR. Specifically, it contains: (1) How much do you currently understand about the COPD progression (i.e., acute exacerbations, disease progression, and the main disease outcome trajectory)? (Perceived susceptibility and severity). (2) Please share your thoughts and feelings about participating in PR exercises or harmonica therapy (Perceived benefits). (3) Could you discuss the difficulties and obstacles you encountered while adhering to PR therapy? (Perceived barriers). (4) What motivates you to maintain regular exercise and harmonica playing? (Self-efficacy and cues to action). Before the official interview started, the interviewer introduced its purpose, stressed confidentiality, and recorded the process after obtaining consent. Interviews were recorded via online video, and field notes captured contextual details and non-verbal cues. After the interview, the recordings were sorted, and the interview content was transcribed verbatim into text. Preliminary classification and coding were then conducted.

### 2.6 Data analysis

The data were analyzed using a deductive thematic analysis approach, following Braun and Clarke’s six-phase framework (36, 37). This involved (1) familiarizing ourselves with the data, (2) generating initial codes based on predefined theoretical constructs, (3) searching for themes, (4) reviewing themes, (5) defining and naming themes, and (6) producing the final report. To ensure the reliability of the analysis, two researchers, Q. Zeng and X. Yang, independently coded the data and identified initial themes. Any discrepancies in coding were resolved through discussion, with the process documented, and experts were consulted when necessary, until a consensus was reached. One example of this process is when Q. Zeng initially coded “emotional distress and anxiety” as a sub-theme under “perceived threats,” X. Yang categorized it as a sub-theme related to “perceived barriers.” After discussion, we agreed to classify it as a sub-theme under “perceived threats,” supported by participant quotes highlighting how emotional challenges, such as distress and anxiety, were anticipated to arise from failing to adhere to home-based PR. This process ensured that the final themes accurately reflected the participants’ experiences and perspectives.

Significant statements related to patient adherence to home-based PR were extracted, and meanings were formulated from these statements. These meanings were then organized into categories and further grouped into subthemes corresponding to the five dimensions of the HBM: perceived susceptibility and severity, perceived benefits, perceived barriers, cues to action, and self-efficacy. The themes were integrated into a comprehensive description of the factors influencing adherence, identifying the core structures that underpin patient behavior.

### 2.7 Ethical considerations

The study received approval from the Ethics Committee of the First Hospital of Guangzhou Medical University (No. ES-2023-075-01). All participants provided informed consent, and confidentiality was maintained throughout the research process.

## 3 Results

We interviewed 18 patients (77.8% male), aged 39–70 years (mean = 58.78, standard deviation = 9.37). Ten were retirees (55.6%), six were employed (33.3%) and two were unemployed (11.1%). Seven had a monthly income under 4,000 yuan (38.9%). Ten had severe COPD, with a mean forced expiratory volume in one second predicted of 52.3 ± 24.3%. Education levels included eight high school, five undergraduate, and five with junior high or less. This diverse group provides a solid data foundation for analysis. Interviews averaged 56 min (range: 40–80). Guided by HBM theory, we identified 2 themes, 5 sub-themes, 28 categories, and 151 codes (Table 1). Additional details are in Table 2.

**Table 1:** Summary of themes and sub-themes extracted from the transcripts.

Themes	Sub-themes	Categories
Facilitating health beliefs	Perceived threats (perceived susceptibility and perceived severity)	Risk of mobility decline and increased dependence
		Risk of health deterioration and functional impairment
		Emotional distress and anxiety
		Fear of acute exacerbations and disease uncertainty
		Risk of increased financial strain
		Risk of social isolation and estrangement
	Perceived benefits	Enhanced physical health
		Improved daily functionality and independence
		Enhanced physical strength and endurance
		Enhanced psychological well-being and self-confidence
		Enhanced social engagement and reduced isolation
		Decreased medical dependence and healthcare utilization
	Cues to action	Self-awareness of current function decline
		Peer influence and social modeling
		Attention-seeking and social reinforcement
		Vicarious motivation from success stories
		Environmental cues
	Self-efficacy	Confidence in health management
		Confidence through social reconnection
		Sense of accomplishment and positive reinforcement
		Mental resilience and religious beliefs
Challenges	Perceived barriers	Physical limitations and exercise-induced fatigue
		Emotional distress and psychological barriers
		Perceived ineffectiveness and exercise monotony
		Time constraints and caregiving responsibilities
		Lack of family support and social assistance
		Cost-related barriers to pulmonary rehabilitation
		Environmental limitations and poor exercise conditions

**Table 2:** Demographics and characteristics of participants (*n* = 18).

Participants ID	Gender	Age	Marital status	Highest education level attained	Residential type	Occupation status	Monthly income, CNY	Medical insurance type	FEV_1_% predicted value	Interview time, minutes
Patient 1	Female	39	Married	College degree	Urban	Employed	2001–4,000	Public health insurance	58.0%	64
Patient 2	Male	62	Married	Bachelor degree above	Urban	Retired	4,001–6,000	Public health insurance	26.0%	60
Patient 3	Male	62	Married	Bachelor degree above	Urban	Retired	6,001–8,000	Public health insurance	37.1%	42
Patient 4	Male	57	Married	College degree	Urban	Unemployed	2001–4,000	Urban medical insurance	109.9%	80
Patient 5	Male	66	Married	College degree below	Urban	Retired	2001–4,000	Public health insurance	38.0%	72
Patient 6	Male	70	Married	Junior high school degree	Urban	Retired	<2000	Urban medical insurance	29.2%	42
Patient 7	Male	66	Widowed	Junior high school degree	Urban	Unemployed	2001–4,000	Urban medical insurance	33.7%	59
Patient 8	Female	45	Married	Bachelor degree above	Urban	Employed	6,001–8,000	Public health insurance	58.2%	56
Patient 9	Male	69	Single	College degree	Urban	Retired	>8,001	Public health insurance	79.7%	40
Patient 10	Male	64	Married	Bachelor degree above	Urban	Retired	4,001–6,000	Public health insurance	39.4%	54
Patient 11	Male	52	Married	College degree	Urban	Employed	>8,001	Urban medical insurance	93.0%	51
Patient 12	Female	65	Married	Junior high school degree	Urban	Retired	4,001–6,000	Public health insurance	75.4%	52
Patient 13	Male	52	Married	College degree	Urban	Employed	6,001–8,000	Urban medical insurance	48.2%	54
Patient 14	Male	61	Married	Primary school degree below	Rural	Retired	<2000	Rural medical insurance	26.7%	66
Patient 15	Female	50	Married	College degree	Urban	Employed	<2000	Commercial insurance	60.1%	46
Patient 16	Male	68	Married	College degree	Urban	Retired	6,001–8,000	Urban medical insurance	34.9%	57
Patient 17	Male	65	Married	College degree	Urban	Retired	4,001–6,000	Urban medical insurance	61.8%	46
Patient 18	Male	45	Married	Primary school degree below	Rural	Employed	4,001–6,000	Rural medical insurance	31.2%	67

FEV_1_%, forced expiratory volume in one second.

### 3.1 Theme 1: Facilitating health beliefs

This theme encompassed participants’ beliefs about their health and the value of home-based PR, which significantly influenced their adherence behaviors. Within this theme, we identified four sub-themes: perceived threats, perceived benefits, cues to action, and self-efficacy. These sub-themes reflected the core constructs of the HBM and highlighted the psychological and social factors that motivated participants to adhere to home-based PR.

#### 3.1.1 Sub-theme 1: perceived threats (perceived susceptibility and perceived severity)

This theme highlights the various threats to participants’ health and well-being if they do not adhere to home-based PR program. They primarily feared three outcomes: declining mobility and increased dependence, leading to deteriorating physical function; significant overall health deterioration and functional impairment; and acute exacerbations with disease uncertainty. These primary threats were expected to result in secondary consequences, including heightened emotional distress and anxiety, increased financial burdens from more frequent hospitalizations, and social isolation due to reduced social engagement. Overall, these perceived threats encompass both direct health-related consequences and subsequent emotional, financial, and social challenges resulting from non-adherence to the PR program.

##### 3.1.1.1 Risk of mobility decline and increased dependence

Participants perceived a significant threat to a potential decline in their daily functioning and independence if they did not persist with their physiotherapy exercises. They might face greater difficulties in daily life and become more dependent on assistive devices, such as wheelchairs.

“If I do not keep training, I might find difficult to keep the ability to do simple things like cooking or dressing myself in future” (Patient 11).

“Not taking action to improve my breathing could make it harder to walk independently, and I might eventually need a wheelchair” (Patient 2).

##### 3.1.1.2 Risk of health deterioration and functional impairment

Participants believed that not maintaining their rehabilitation regimen could lead to worsening lung function, increased reliance on medical interventions like oxygen, and possibly severe outcomes, such as the need for a lung transplant.

“Without keeping up with my exercise routine, my lungs will weaken, and I might end up needing a lung transplant” (Patient 5).

“I can already feel my breathing worsening. If I do not stick to the rehabilitation, I might be dependent on oxygen all the time” (Patient 7).

##### 3.1.1.3 Emotional distress and anxiety

Participants anticipated that failing to adhere to PR program would lead to emotional distress and heightened anxiety. The uncertainty surrounding the progression of their condition contributed to feelings of vulnerability and emotional strain.

“I feel overwhelmed by the thought of how much worse my breathing could become” (Patient 4).

“Anxiety overwhelms me in such situations. If it gets worse, I will not be able to care for myself or go to the hospital” (Patient 10).

##### 3.1.1.4 Fear of acute exacerbations and disease uncertainty

Participants sensed that failing to follow their rehabilitation plan could make their disease progression unpredictable, increasing their fear of acute exacerbations.

“When I was in the hospital before, I saw patients who were admitted for acute illnesses due to worsening COPD. They deteriorated so quickly that it really scared me. I’m afraid that if I do not listen to the doctors, I’ll end up like them—it’s too frightening to even imagine” (Patient 10).

“I’m terrified that one day, without warning, I could find myself struggling to breathe and not be able to do anything about it” (Patient 6).

##### 3.1.1.5 Risk of increased financial strain

Participants acknowledged that neglecting to keep exercising could lead to increased medical costs, loss of work capacity, and a negative effect on their family’s financial stability.

“Dependence on medication is something I do not want. Failing to take rehabilitation seriously will only lead to higher costs for hospital visits and treatments” (Patient 5).

“I am the head of the family. If I lose my ability to work because of this disease, it will definitely have a significant impact on the family” (Patient 11).

##### 3.1.1.6 Risk of social isolation and estrangement

Participants considered that not adhering to their exercise routines could lead to physical limitations and increased symptoms, such as loss of mobility and frequent coughing. This, in turn, could result in reduced social integration, with others potentially avoiding them due to misconceptions about their health.

“Not going to therapy, not doing rehabilitation exercises—I might not even be able to walk and will have to lie down at home” (Patient 7).

“Coughing often can make others, who do not understand my condition, worry that it’s contagious and lead them to avoid me at times” (Patient 9).

#### 3.1.2 Sub-theme 2: perceived benefits

This theme reflected participants’ perceptions of the potential or actual benefits they associate with continuing the PR program. Many participants reported enhanced physical health, feeling better overall with fewer complications. They also noted improved daily functionality and independence, able to perform tasks without help, and anticipated enhanced physical strength and endurance for more strenuous activities. These physical improvements were linked to enhanced psychological well-being and self-confidence, as participants felt more optimistic about managing their health. Socially, they experienced enhanced social engagement and reduced isolation were common, becoming more involved in family and community activities. Finally, participants perceived continued adherence to the PR program would lead to decreased medical dependence and healthcare utilization, fostering a more independent lifestyle. These perceived benefits reinforced their commitment to the rehabilitation program.

##### 3.1.2.1 Enhanced physical health

Participants reported improvements in their physical health as a result of continued PR program. They noted a reduction in the frequency of illnesses, such as fewer colds, and experienced a general sense of physical improvement, including weight gain, indicating overall better health.

“I used to catch a cold once a month, but now, after exercising, I have not caught a cold for two months” (Patient 6).

“After this period of exercise, I’ve gained a little weight” (Patient 7).

##### 3.1.2.2 Improved daily functionality and independence

Participants experienced the positive outcomes of consistent participation in PR program in terms of daily functionality and independence. They reported a reduced need for medical assistance, such as oxygen use, and improved endurance, which allowed them to perform activities like cleaning their home without fatigue.

“Originally, I needed to use oxygen after bathing, but now I do not necessarily need it immediately afterward” (Patient 2).

“I do not get tired easily now. I can mop my 100-square-meter house in one go” (Patient 1).

##### 3.1.2.3 Enhanced physical strength and endurance

Participants recognized that consistent engagement in PR program significantly improved their physical strength and endurance. They noticed a marked increase in their ability to perform physical activities, such as walking longer distances without breaks or carrying out tasks like holding a child, which they had previously found difficult. This improvement contributed to a greater sense of capability and physical well-being.

“Now, I can cross the overpass in one go, from the steps to the top, without having to take a break every time I walk a certain distance” (Patient 3).

“I can hold my granddaughter now, which I could not do before. Recently, I’ve been able to hold her for a while and carry her around for walks” (Patient 10).

##### 3.1.2.4 Enhanced psychological well-being and self-confidence

Participants acknowledged that continuing with PR program had a positive impact on their mental health and self-confidence. They experienced reduced anxiety, improved emotional outlook, and a greater sense of self-assurance, recognizing that these programs contributed to both physical and mental improvement.

“Practicing for a while, I noticed a positive effect, which boosted my confidence and made me feel better” (Patient 12).

“After more than three months of exercise, I feel that both my physical and mental outlook have improved. I was a bit anxious before, but now I feel better overall, and I’m in a better state than before” (Patient 9).

##### 3.1.2.5 Enhanced social engagement and reduced isolation

Participants noted that staying consistent with PR program reduced their fatigue, making it easier for them to engage in social interactions. They reported being able to go out for tea more often and join family outings and social events, which had previously been difficult due to exhaustion. This improved energy and participation in social activities contributed to a richer social life, reducing feelings of isolation and enhancing their overall well-being.

“Rehabilitation exercises help me feel less tired, allowing me to go out for tea more often” (Patient 14).

“By keeping up with exercise, I’ll be able to join my family for outings and social events again, instead of staying home feeling too tired” (Patient 5).

##### 3.1.2.6 Decreased medical dependence and healthcare utilization

Participants observed that regular PR program reduced their reliance on medical treatments, such as hospital visits and medications. They experienced fewer symptoms, like persistent coughing and phlegm production, which led to a decreased need for medications and medical interventions.

“I used to need to go to the hospital for an infusion every time I caught a cold, but after exercising, I have not needed to go to the hospital for an infusion during the last two colds” (Patient 6).

“It was difficult to cough up phlegm before, and I had to take medicine to reduce it. However, after exercising over the past two months, I’ve rarely needed medication. Although I still have a slight cough and occasionally produce phlegm, it’s much easier to cough up than before. I only need to cough two or three times to clear it” (Patient 5).

#### 3.1.3 Sub-theme 3: cues to action

Participants identified several cues that prompted adherence to the home-based PR program. Internal cues included self-awareness of declining physical function, motivating them to prioritize PR and initiate changes. External social cues involved peer influence and social modeling, encouraging adherence to maintain their standing among peers. Additionally, attention-seeking and social reinforcement from family and friends made participants feel cared for and supported, fostering a desire to continue. Success stories highlighting others’ progress also inspired adherence. External environmental cues, such as reminders from healthcare providers and the availability of exercise equipment, acted as triggers to sustain commitment to the PR regimen. These internal and external factors—self-awareness, social influence, and environmental prompts—collectively sustained participants’ engagement with the rehabilitation program.

##### 3.1.3.1 Self-awareness of current function decline

Awareness of declining function prompts participants to persist in PR program. Recognizing symptoms such as discomfort or difficulty breathing motivates them to engage in exercise training, as they seek improvement and better management of their condition.

“The more uncomfortable I feel, the more I feel the urge to play the harmonica and think about how I can improve myself” (Patient 2).

“When I have difficulty breathing, as I’ve been experiencing lately, I feel motivated to exercise” (Patient 8).

##### 3.1.3.2 Peer influence and social modeling

Peer influence motivated participants to continue engagement in PR program. Observing others complete their workouts or perform excellently, such as playing the harmonica, created a sense of urgency or aspiration, prompting participants to maintain their own exercise routines.

“When I see that others have already finished their workout, not working out myself makes me feel left behind, so I feel compelled to start immediately” (Patient 5).

“I saw someone playing the harmonica really well in a video, and I want to be able to do the same” (Patient 18).

##### 3.1.3.3 Attention-seeking and social reinforcement

Attention-seeking and social reinforcement, such as sharing exercise progress in a group or receiving reminders from others, provided motivation for participants to persist with PR program. These external cues fostered a sense of accountability and support, encouraging continued participation.

“I post videos of my daily exercises and harmonica playing in the group, and it makes me feel like someone cares about me, which motivates me to keep going” (Patient 3).

“My colleague would remind me whenever we exercised, and I would go and exercise” (Patient 1).

##### 3.1.3.4 Vicarious motivation from success stories

Success stories from others inspired participants to persist with their PR program. Hearing about the positive changes experienced by fellow patients encouraged them to remain committed to their own exercise routines.

“Meeting another patient at the rehabilitation center, who spoke about how rehabilitation changed his life, motivated me to continue with my exercise routine” (Patient 11).

##### 3.1.3.5 Environmental cues

Environmental cues, such as seeing exercise equipment, serve as reminders and triggers to continue PR program.

“As soon as I saw the elastic band, I felt the urge to exercise” (Patient 1).

#### 3.1.4 Sub-theme 4: self-efficacy

Participants reported several factors that will impact their self-efficacy in adhering. Confidence in health management was a key, stemming from an enhanced sense of control due to increased perceived ability in disease management built through the PR program. Additionally, confidence through social reconnection referred to the enhanced confidence gained from improved health conditions, allowing them to reconnect with their social relationships. Many participants also reported a sense of accomplishment and positive reinforcement constantly boosted their confidence. Finally, mental resilience and personal beliefs were also mentioned, participant’s determination and perseverance to overcome challenges and their religious belief boosted their self-efficacy for adherence.

##### 3.1.4.1 Confidence in health management

Confidence in health management emerged as a key motivator for participants to persist in PR program. Feeling more in control of their health and recognizing that consistent effort in rehabilitation directly improved their condition strengthened their belief in their ability to manage their health effectively. This sense of control encouraged continued commitment to the program.

“Feeling more in control of my health now, sticking to my rehabilitation exercises helps me manage my condition more effectively” (Patient 9).

##### 3.1.4.2 Confidence through social reconnection

Rejoining social life gave participants the confidence to persist in PR program. Experiencing less fatigue during social outings reinforced the belief that consistent effort leads to improved physical capacity, fostering a sense of self-efficacy and motivating ongoing exercise.

“Last week, going out with my friends, I did not feel as tired as I used to. It showed me that keeping at it helps me keep up” (Patient 15).

##### 3.1.4.3 Sense of accomplishment and positive reinforcement

Receiving positive feedback and witnessing progress boosted participants’ confidence to stay committed to PR program. Compliments from instructors and improvements in medical indicators reinforced their belief in their ability to improve, fostering a sense of accomplishment and motivating continued effort.

“Later, as I gradually improved, the teacher said that my performance was up to standard, which made me happy” (Patient 5).

“The doctor who examined me each time also reviewed the report and said that my results were slightly better than last time. This made me feel more confident” (Patient 9).

##### 3.1.4.4 Mental resilience and religious beliefs

Mental resilience and personal beliefs contributed significantly to the confidence needed to continue PR program. Despite experiencing fatigue and exhaustion, the belief in the necessity of perseverance drove participants to push through challenges. Personal beliefs, such as faith, also offered a sense of strength, helping to overcome reluctance and maintain commitment to the rehabilitation process.

“I feel exhausted every time I start doing it, but I think I need to keep going” (Patient 10).

“Christianity will help me regain my confidence and overcome my laziness” (Patient 1).

### 3.2 Challenges

This theme highlighted the barriers that hindered participants’ adherence to home-based PR. These challenges reflected the practical and psychological obstacles that participants faced, which limited their ability to consistently engage in the PR program. Within this theme, we identified one sub-theme: perceived barriers. This sub-theme encompassed a range of factors that negatively impacted participants’ adherence behaviors.

#### 3.2.1 Sub-theme: perceived barriers

This theme reflected participants’ perceptions of the barriers hindering their adherence to the PR program. Physical limitations and exercise-induced fatigue were major obstacles, with many participants anticipating discomfort or exhaustion that would prevent exercise completion. Emotional distress and psychological barriers, including frustration and anxiety, also affected motivation to continue. Some participants perceived the exercises as monotonous or ineffective, leading to doubts about their usefulness and disengagement. Time constraints and caregiving responsibilities made it challenging to balance rehabilitation with daily tasks. Lack of family support and social assistance contributed to feelings of isolation, making it harder for them to stay committed. Cost-related barriers were also perceived as limiting access to rehabilitation services, while environmental limitations and poor exercise conditions in some settings further hindered exercises effectively.

##### 3.2.1.1 Physical limitations and exercise-induced fatigue

Participants identified physical limitations, such as low blood oxygen levels, fatigue, and weakness during certain conditions (e.g., menstruation or low blood pressure), as barriers to continuing their PR program. These factors decreased their motivation and often required them to stop or modify their exercise routines.

“When I was doing the fourth task, my blood oxygen saturation dropped, and my heart rate increased, so I could not continue and had to stop” (Patient 2).

“During my menstrual period, I feel weaker. Additionally, sometimes I feel tired when I have low blood pressure. In these situations, I really lack the motivation to exercise” (Patient 1).

##### 3.2.1.2 Emotional distress and psychological barriers

Participants reported that negative emotions, often triggered by external factors such as family interactions or personal stress (e.g., financial losses or family conflict), hindered their motivation to continue with PR program. These emotional downturns reduced their willingness to engage in physical activity.

“Sometimes it’s inexplicable, and other times it may be triggered by certain comments from family members, such as things my husband says, which can affect my mood. I feel down and not in the best mood, which also reduces my willingness to exercise” (Patient 5).

“Sometimes, when I encounter something upsetting, such as the stock market falling or being scolded by my wife and children, I feel depressed and unwilling to move” (Patient 4).

##### 3.2.1.3 Perceived ineffectiveness and exercise monotony

Participants expressed that the repetitive nature of the exercises led to a sense of monotony and boredom, which diminished their interest and motivation to continue. They felt that the lack of noticeable progress or change made it difficult to sustain their commitment to the rehabilitation program.

“At first, I was a little interested, felt good, and had fun. But after repeating the same thing every day, it started to feel monotonous and boring” (Patient 14).

“I have been exercising for a while, but I found that the results are always the same, which makes me unwilling to continue” (Patient 18).

##### 3.2.1.4 Time constraints and caregiving responsibilities

Participants highlighted that their busy schedules and family responsibilities, such as work commitments and caregiving duties, made it difficult to find time for PR program. These time constraints often hindered their ability to consistently engage in the program.

“I trade stocks, so time is tight, and I usually do not have time during the day” (Patient 4).

“I did all the cleaning and laundry. I have two grandkids and children, but we do not live together. I have to drive to their place, and it’s a 40-min round-trip, so it takes a lot of time” (Patient 17).

##### 3.2.1.5 Lack of family support and social assistance

Participants expressed that a lack of attention and support from family members was a significant barrier to their PR program. As family members were preoccupied with their own responsibilities, patients felt isolated and overwhelmed, especially when they needed help or understanding during difficult times.

“No one in the family noticed, and I did not say anything. They did not care about these things because they were busy with their own work and family matters. No one noticed or was concerned about my situation” (Patient 15).

“I try to manage everything on my own, but sometimes I feel overwhelmed. My family is busy with their own lives, and they do not really understand the level of support I need. I wish they were more involved, especially when I’m feeling exhausted or unwell” (Patient 13).

##### 3.2.1.6 Cost-related barriers to pulmonary rehabilitation

Participants mentioned the financial strain caused by the costs associated with PR program, including transportation and accommodation expenses. For some, these additional costs were a significant barrier to consistently attending rehabilitation sessions.

“My home is still far from the city where the rehabilitation center is located, and the accommodation and transportation costs for my trips there are quite high—almost 1,000 yuan” (Patient 8).

##### 3.2.1.7 Environmental limitations and poor exercise conditions

Participants reported environmental challenges that hindered their ability to engage in PR program. Factors such as limited space at home and unfavorable environmental conditions, like high altitudes and poor air quality, were identified as barriers to consistent exercise. These external conditions significantly impacted their ability to complete rehabilitation tasks effectively.

“Because there’s so much stuff piled up in the house, the space is very cramped, making it difficult for me to do my rehabilitation exercises” (Patient 2).

“I went back to Qinghai (anther city) for a while. The altitude there is 2,200 meters (7,200 feet), which is quite high, and the air is thin and lacks oxygen, making it much harder for me to exercise” (Patient 3).

## 4 Discussion

This study is, to our knowledge, the first to use HBM to identify the facilitators and barriers to COPD patients’ adherence to home-based PR. Our aim is to highlight factors that influence adherence, providing insights to help policymakers and service providers develop effective strategies that reduce healthcare burdens and improve quality of life. Within the framework of two main themes (facilitating health beliefs and challenges) and five subthemes (perceived threats, perceived benefits, cues to action, self-efficacy and perceived barriers), we identified 28 categories, including 21 that facilitated adherence and 7 that acted as barriers. Among these, 11 facilitators and one barrier were newly discovered. Notably, we found for the first time that perceived threats, such as physical limitations and fatigue, emotional distress, perceived ineffectiveness, time constraints and caregiving responsibilities, lack of support, cost, and poor exercise conditions, can facilitate adherence to home-based PR. Additional facilitators included reduced medical dependence, self-awareness of functional decline, attention-seeking behaviors, environmental cues, and mental resilience and religious beliefs. These findings underscore the importance of monitoring, support, and reinforcement to promote adherence, highlighting both health and cost-related benefits of home-based PR. The new barrier identified in our study was the impact of environmental limitations and poor exercise conditions, emphasizing the need to consider environmental factors—such as space and exercise conditions—when designing home-based PR programs to improve adherence and health outcomes for COPD patients.

### 4.1 Facilitating health beliefs: facilitators of adherence to home-based pulmonary rehabilitation

Within the theme of facilitating health beliefs, we identified several facilitators confirmed by previous research that can motivate participants to adhere to home-based PR, including perceived benefits [enhanced physical health, improved daily functionality and independence, increased physical strength and endurance (38), psychological well-being and self-confidence (39), enhanced social engagement and reduced isolation (39)], cues to action [peer influence and social modeling, vicarious motivation from success stories (22, 39)], and self-efficacy [confidence in health management, confidence through social reconnection, and a sense of accomplishment with positive reinforcement (40)]. Additionally, our study revealed new facilitators related to perceived threats, such as physical limitations and fatigue, emotional distress, perceived ineffectiveness, time constraints and caregiving responsibilities, lack of support, cost, and poor exercise conditions. Other newly identified facilitators included reduced medical dependence, self-awareness of functional decline, attention-seeking behaviors, environmental cues, and mental resilience and religious beliefs.

Our study found that participants’ perceived improvements in physical health, daily functioning, strength, and psychological well-being were key factors in sustaining adherence to home-based PR. Home-based PR had been shown to reduce symptom severity (measured by CAT score) by 4.6 points and improve dyspnea (mMRC scale) by 0.73 points in patients with COPD (41). For older adults COPD patients, enhanced physical function increases independence in daily activities, boosting recovery motivation (42), while symptom relief improves psychological well-being by restoring health control and reducing anxiety (43). Therefore, implementing mobile health tools for progress tracking and positive reinforcement can enhance these benefits, providing a cost-effective chronic disease management solution for aging populations.

Social engagement, reduced isolation, and peer influence were also crucial for maintaining home-based PR adherence. Older adults often face the dual challenges of shrinking social networks and declining physical health (44). Home-based PR significantly improves exercise capacity, increasing endurance shuttle walk test time by 340 s (95% CI, 153–526) in older adults COPD patients (45). These physical improvements directly enhanced social participation, reducing isolation and enriching their social lives (46). This positive impact was further reinforced in group interactions, where participants observed others’ adherence and progress, motivating their own active participation. Peer influence, driven by the observational learning, created a virtuous cycle, reinforcing sustainable rehabilitation. Thus, home-based PR programs should incorporate diverse social support systems, leverage community resources, and foster peer motivation. This approach addresses patients’ psychological and social needs while tackling public health challenges in China’s aging society.

Several factors that enhance self-efficacy in adhering to home-based PR were identified, including confidence in managing their health, the confidence gained through social reconnection, and a sense of accomplishment through positive reinforcement. A key strength of home-based PR is its convenience, which allows patients to exercise in a familiar environment while receiving professional support through remote monitoring and guidance (47, 48). This support helps patients gain disease management and rehabilitation skills, boosting their confidence in managing their health. As their skills improved, patients became more willing to engage in social activities, which not only alleviated social isolation but also reinforced their confidence in recovery through peer support (49, 50). In line with self-efficacy theory, additional sources such as mastery experiences, where patients notice personal progress in managing symptoms, and vicarious experiences, such as observing others succeed in rehabilitation, also contribute to adherence (51). Furthermore, verbal encouragement from healthcare providers and emotional states (e.g., stress or fatigue) play crucial roles in shaping patients’ confidence in their ability to stick with the program (52). Therefore, home-based PR should be integrated into primary health services, training community healthcare professionals for ongoing support. The community should also create a support network that encourages social participation. This patient-centered approach not only enhances COPD recovery outcomes but also reduces healthcare system burdens.

An unexpected finding was that factors like decreased mobility, health deterioration, emotional distress, financial strain, and social isolation influenced persistence in home-based PR. Severely affected COPD patients, motivated to alleviate dyspnea, improve fitness, and reduce loneliness, were more likely to engage in home-based PR, consistent with Baiardini’s findings on symptom impact and treatment awareness (53). In China, where COPD burden is high (54), patients may also view home-based PR as a cost-effective way to reduce medical expenses, further motivating adherence. To address these challenges, personalized programs targeting psychological and social needs—such as counseling, family involvement, and community activities—are essential. Low-cost support models, including government subsidies or charitable funding, can ease financial pressures and improve accessibility, promoting both adherence and efficient resource allocation.

Another novel finding was the reduction in medical dependence and healthcare utilization, which facilitated adherence to home-based PR. As participants, health improved, they reported fewer medical visits. In China, the uneven distribution of medical resources due to regional development imbalances (26) makes healthcare access particularly limited. Home-based PR offers an essential alternative for managing the condition effectively at home. With tailored exercises and self-management, patients experience fewer exacerbations and hospital visits. The suggests that promoting home-based PR as a core strategy for chronic disease management—especially in resource-limited areas—along with telemedicine and community support services, can further reduce healthcare utilization and enhance patients’ adherence to rehabilitation.

Self-awareness of current function decline was identified as another novel facilitator in promoting adherence to home-based PR. This awareness appears to be a critical competency that empowers patients to take an active role in their care (55). Research indicates that 69% of COPD patients seek medical attention during an exacerbation (56), a finding corroborated by our participants who reported increased motivation to engage in home-based PR when they noticed declines in their physical abilities or experienced discomfort. This self-awareness served as a powerful motivator for consistent participation. To capitalize on this finding, we recommend integrating self-assessment tools (e.g., symptom diaries or mobile apps) for real-time condition monitoring and self-management. Educating patients about disease progression further enables proactive measures. These strategies enhance patients’ sense of control and offer a humanized approach to chronic disease management in aging societies.

Attention-seeking and social reinforcement, acting as cues to promote adherence to home-based PR, are new facilitators identified in our study. Nearly one in six adults with COPD experiences social isolation (57) due to physical limitations. Participants in our study were able to share video workouts in a WeChat group, gaining attention and recognition from others. This positive social feedback significantly enhanced their motivation for recover. Unlike hospital-based rehabilitation, home-based PR lacks direct medical staff supervision. However, social reinforcement through virtual communities creates a continuous supportive environment. These external cues foster a sense of community and encouragement, both of which are essential for long-term adherence to rehabilitation. For future home-based PR programs, we recommend incorporating social features, such as mobile apps for progress sharing, reminders, and peer feedback, alongside community activities like rehabilitation demonstrations or health talks, to enhance social participation and achievement.

Environmental cues, such as visual reminders, offer a novel way to promote adherence to home-based PR. Visual aids have been shown to improve compliance (58). In our study, elastic bands served as an intuitive visual and tactile cue, effectively reminding patients to perform rehabilitation exercises. The presence of exercise equipment in the home environment reinforces the importance of rehabilitation and encourages routine participation. Older COPD patients, especially those with memory loss, can benefit from these cues by reducing forgetfulness, preventing procrastination, and improving adherence. To maximize home-based PR effectiveness, low-cost tools (e.g., elastic bands) and visual materials (e.g., posters, videos) should be prioritized. Integrating these with community health education can expand PR’s reach, particularly in rural areas, offering an economical and practical solution.

In conclusion, these findings highlight critical strategies for optimizing home-based PR in aging populations. By addressing physical, psychological, and social barriers while leveraging facilitators like self-awareness and environmental cues, healthcare systems can enhance adherence and reduce the burden of COPD. These approaches offer scalable, cost-effective solutions to improve chronic disease management and support healthy aging in resource-limited settings.

### 4.2 Challenges: barriers to adherence in home-based pulmonary rehabilitation

Within the theme of Challenges, we identified six barriers’ factors that have been previously reported: physical limitations and exercise-induced fatigue (38, 59), emotional distress (59), perceived ineffectiveness and exercise monotony (22), time constraints (60), lack of family support (38), and cost-related barriers to PR (61). The new barrier identified in our study was the impact of environmental limitations and poor exercise conditions.

Participants reported challenges such as physical limitations, exercise-induced fatigue, and emotional distress, all of which hinder adherence to home-based PR. Those with poorer lung function in our study were more likely to report these barriers, consistent with studies linking dyspnea, anxiety, and reduced exercise capacity to PR discontinuation (59). Patients with severe COPD struggle with increased shortness of breath, reduced stamina, and muscular weakness, making exercise more difficult. A study using wearable monitors found that SpO2 and heart rate changes during exercise strongly correlate with lung function, highlighting the need for adequate oxygen support (62). These physical challenges, combined with emotional distress linked to physical inactivity (63), further discourage participation. These findings underscore the need for age-friendly, accessible PR programs that address the unique challenges faced by older adults with COPD. Integrating personalized exercise plans, real-time oxygen monitoring, and psychological support can reduce barriers to adherence, improve functional independence, and alleviate the growing burden of chronic respiratory diseases in the aging population.

Another significant barrier to adherence was the cost of PR, particularly the financial strain caused by transportation and accommodation expenses. In China, where regional disparities in healthcare are stark and high-quality medical resources are under-allocated in remote areas (26), traveling to urban centers for PR programs often incurs substantial costs for transportation and lodging, which can be prohibitive for low-income families. These financial pressures discourage adherence to rehabilitation programs. From a public health perspective, this finding underscores the urgent need for cost-effective, scalable solutions. Policymakers should prioritize strategies to reduce these barriers, such as providing transportation subsidies, establishing regional rehabilitation hubs, and integrating home-based PR into community health initiatives. Addressing these challenges can improve accessibility for aging populations, reduce disparities in care, and alleviate the long-term economic burden of COPD management on China’s healthcare system.

Additionally, the perceived ineffectiveness of exercises and the monotony of routines pose another key barrier. A cross-sectional study of 1,138 COPD patients found that many have limited knowledge of rehabilitation and poor perceptions of its necessity (64). This lack of awareness can lead patients to doubt the effectiveness of exercise, particularly when they do not experience immediate improvements. Such doubts can make daily activities feel monotonous, further diminishing motivation and hindering engagement with the program. In this context, personalized education and targeted interventions that highlight the incremental benefits of exercise could play a crucial role in improving adherence. Public health initiatives should focus on increasing awareness of rehabilitation programs and providing continuous support, particularly for aging populations, to reduce misconceptions and increase participation.

Time constraints and caregiving responsibilities were significant barriers to adhere to home-based PR, particularly for those with caregiving duties. In China, many older adults individuals assist in caring for younger generations or grandchildren (65). These responsibilities limit the time and energy available for PR exercises. As family obligations take precedence, patients may find it difficult to prioritize their own health needs, including consistent participation in home-based PR. Public health interventions should therefore consider integrating caregiving support into rehabilitation programs. This could include providing resources to help older adults caregivers balance their roles while managing their own health, as well as offering flexible rehabilitation schedules. Additionally, initiatives to educate families on the importance of self-care for caregivers could help reduce the burden on older adults and enhance their engagement in rehabilitation.

A lack of family support and social assistance was another common barrier to sustained participation in home-based PR, especially for older adults COPD patients. With an aging population and the rise of empty-nest households in China (25), family members may not always be available to provide the necessary assistance. The absence of adequate social support isolates COPD patients, making it harder for them to stay committed to PR exercises. This underscores the need for community-based interventions to provide support in the absence of family. For instance, mHealth interventions improved self-care and adherence through enhanced social support (66), while family health reduced frailty by promoting health literacy and healthier behaviors (67). Strengthening family health and creating local support systems, potentially supplemented by digital tools, could alleviate this isolation, helping older adults patients maintain adherence to rehabilitation programs and improve their overall health outcomes.

An important new barrier identified in our study was the impact of environmental limitations and poor exercise conditions. In home-based PR, COPD patients often rely on their home environment for exercise, but many faces inadequate spaces or safety concerns (68). In China, where urbanization and housing conditions vary widely, many older adults COPD patients live in crowded or substandard housing, limiting their ability to perform rehabilitation exercises effectively. Additionally, challenging climate conditions or environment factors can lead to reduced motivation, making it difficult for patients to maintain consistent exercise routines (69). Addressing these environmental challenges by improving home exercise conditions and offering guidance on creating suitable spaces for physical activity can enhance adherence to home-based PR, leading to better health outcomes for COPD patients.

These findings offer valuable insights into the factors that hinder adherence to home-based PR. Addressing these barriers—particularly the environmental limitations—could significantly improve patient engagement and the effectiveness of rehabilitation programs. Future research should explore ways to integrate home-based PR into community health strategies offers a scalable solution to support healthy aging and reduce the burden of COPD in resource-limited settings.

### 4.3 Implications for practice

The findings from this study highlight several key strategies for improving adherence to home-based PR, particularly important in the context of an aging population and the rising burden of chronic diseases like COPD. Rehabilitation programs must address both physical and emotional needs, with exercises tailored to individual capabilities and psychological support to sustain engagement. Given the physical decline and mental health challenges common among older adults, these elements are crucial. Peer support and social modeling can foster a sense of community, helping reduce social isolation—a significant issue in aging populations. Self-monitoring tools, such as apps or tracking systems, can keep patients engaged by visualizing progress and highlighting the impact of non-adherence. Social support, including virtual group interactions and family involvement, is critical to reduce the caregiving burden and reinforce rehabilitation. Environmental factors, such as creating a suitable home exercise space with visual reminders, also support adherence, especially for older individuals who may need cues to stay on track. Lastly, emphasizing the long-term benefits—such as reduced healthcare costs and fewer medical visits—can motivate older patients, addressing both health and economic concerns. These strategies can improve adherence, enhance outcomes, and offer a cost-effective solution to chronic disease management in an aging society.

### 4.4 Strengths

This study has several strengths that enhance its contribution to the field. First, it is one of the first to apply the HBM to explore adherence to home-based PR for COPD patients, offering new theoretical insights into the role of perceived benefits and barriers. Second, the use of a deductive thematic analysis approach, guided by the HBM, allowed us to systematically explore patients’ experiences and perceptions, providing a structured yet nuanced understanding of the facilitators and barriers to adherence. Third, our findings have direct implications for designing patient-centered interventions that address both psychological and practical barriers. Finally, the inclusion of participants from diverse backgrounds enhances the transferability of our findings to a broader population of COPD patients.

### 4.5 Limitations and future directions

This study has several limitations. First, it focused on patients who had participated in home-based PR for at least 3 months, excluding those who did not engage, which may introduce sample bias. Future studies should include those who did not participate or discontinued PR for a more comprehensive view. Second, the interviews were conducted via online video, but some patients chose to turn off their cameras, limiting the ability to capture nonverbal cues. Combining online and face-to-face interviews in future research could enrich data collection. Finally, the interviews were conducted in Chinese or the local Cantonese dialect and later translated into English, which may have led to some loss in meaning. Future studies could use bilingual teams or back-translation techniques to ensure accuracy.

## 5 Conclusion

This study provides valuable insights into the factors influencing adherence to home-based PR within the framework of the HBM. Both facilitating health beliefs (perceived threats, perceived benefits, cues to action, and self-efficacy) and challenges (perceived barriers) play a crucial role in patient engagement. Key facilitators include addressing physical, emotional, and social needs, promoting self-monitoring, and leveraging peer support. Environmental factors also emerged as significant, suggesting that rehabilitation programs should consider home settings and provide guidance for creating conducive exercise environments. Barriers such as physical limitations, time constraints, lack of social support, and environmental challenges must be addressed through personalized, flexible program designs and greater involvement of family members and peers. By recognizing these factors, healthcare providers can enhance patient adherence, improve rehabilitation outcomes, and ultimately, optimize the effectiveness of home-based PR. Continued research is needed to refine strategies that integrate these strategies into public health initiatives can improve accessibility, promote long-term health benefits, and provide a cost-effective solution to managing chronic conditions in an aging society.

## Data Availability

The original contributions presented in the study are included in the article/supplementary material, further inquiries can be directed to the corresponding author.
